# Physical, Psychological and Social Frailty Are Predictive of Heart Failure: A Cross-Sectional Study

**DOI:** 10.3390/jcm11030565

**Published:** 2022-01-23

**Authors:** Izabella Uchmanowicz, Aleksandra H. Pasieczna, Monika Wójta-Kempa, Robbert J. J. Gobbens, Agnieszka Młynarska, Kenneth M. Faulkner, Michał Czapla, Remigiusz Szczepanowski

**Affiliations:** 1 Department of Nursing and Obstetrics, Faculty of Health Sciences, Wroclaw Medical University, 51-618 Wrocław, Poland; izabella.uchmanowicz@umw.edu.pl; 2 Institute of Heart Diseases, University Hospital, 50-566 Wroclaw, Poland; 3 Faculty Accounting and Finance, Kozminski University, 03-301 Warsaw, Poland; 1264-phdf@kozminski.edu.pl; 4 Department of Health Humanities and Social Science, Wroclaw Medical University, 51-618 Wrocław, Poland; monika.wojta-kempa@umw.edu.pl; 5 Faculty of Health, Sports and Social Work, Inholland University of Applied Sciences, 1081 HV Amsterdam, The Netherlands; robbert.gobbens@inholland.nl; 6 Zonnehuisgroep Amstelland, 1186 AA Amstelveen, The Netherlands; 7 Department Family Medicine and Population Health, Faculty of Medicine and Health Sciences, University of Antwerp, 2610 Wilrijk, Belgium; 8 Department of Gerontology and Geriatric Nursing, School of Health Sciences, Medical University of Silesia, 40-055 Katowice, Poland; amlynarska@sum.edu.pl; 9 School of Nursing, Stony Brook University, Stony Brook, NY 11794, USA; faulknke@bc.edu; 10 Laboratory for Experimental Medicine and Innovative Technologies, Department of Emergency Medical Service, Wroclaw Medical University, 51-616 Wroclaw, Poland; 11 Department of Computer Science and Systems Engineering, Wrocław University of Science and Technology, 50-370 Wrocław, Poland; remigiusz.szczepanowski@pwr.edu.pl

**Keywords:** heart failure, frailty, psychosocial factors, demographics

## Abstract

Background: Little is known about frailty among patients hospitalized with heart failure (HF). To date, the limited information on frailty in HF is based on a unidimensional view of frailty, in which only physical aspects are considered when determining frailty. The aims of this study were to study different dimensions of frailty (physical, psychological and social) in patients with HF and the effect of different dimensions of frailty on the incidence of heart failure. Methods: The study used a cross-sectional design and included 965 patients hospitalized for heart failure and 164 healthy controls. HF was defined according to the ESC guidelines. The Tilburg Frailty Indicator (TFI) was used to assess frailty. Probit regression analyses and chi-square statistics were used to examine associations between the occurrence of heart failure and TFI domains of frailty. Results: Patients diagnosed with frailty were 15.3% more likely to develop HF compared to those not diagnosed with frailty (*p* < 0.001). An increase in physical, psychological and social frailty corresponded to an increased risk of HF of 2.9% (*p* < 0.001), 4.4% (*p* < 0.001) and 6.6% (*p* < 0.001), respectively. Conclusions: We found evidence of the association between different dimensions of frailty and incidence of HF.

## 1. Introduction

Heart failure (HF) affects over 64 million people worldwide, and the prevalence is expected to double in the next 40 years [1,2]. Frailty syndrome frequently occurs in patients with HF, with prevalence rates ranging from 15 to 74%, depending on the clinical population and assessment methods [3,4,5]. The prevalence of frailty syndrome increases significantly with greater age. Frailty syndrome is present in only 3.2% of patients aged 65–70 years but is present in 23.1% of patients 90 years and older [6,7,8]. The recent FRAIL-HF study provided evidence that frailty may affect more than 70% of heart failure patients over 80 years of age, emphasizing that older HF patients are particularly susceptible to frailty [9,10]. Furthermore, it is estimated that about 50–70% of older patients who have been hospitalized for acute heart failure have some degree of frailty [11]. Frailty syndrome is associated with higher mortality, frequent hospitalization and diminished quality of life [12,13]. The findings of the FRAIL-HF study provided evidence that frailty syndrome may contribute to greater disability, long-term mortality and more frequent readmissions [14].

Frailty is believed to have a bidirectional effect on cardiovascular disease [15,16]. Although research findings have demonstrated that people with cardiovascular disease are more likely to develop frailty, others have provided evidence that individuals with frailty are more likely to develop cardiovascular disease [15,16]. Therefore, frailty can be considered both a consequence of and a potential risk factor for cardiovascular disease (CVD) [17,18]. For example, a meta-analysis and exploratory meta-regression analysis provided evidence that the odds of cardiovascular disease are higher and that time to mortality is shorter among frail and prefrail older adults when compared to their non-frail counterparts [16]. 

Diagnosis of frailty in patients with HF can be challenging because symptoms of fatigue, dyspnea or sarcopenia are manifestations of both clinical conditions [19]. Frailty and HF share common pathophysiologic mechanisms that have not been fully elucidated, but these shared mechanisms may contribute to common symptoms [14]. Further, frailty often overlaps with older age and multimorbidity [13,20]. Therefore, evaluating common deficits in patients with heart failure for the presence of frailty symptoms may prove valuable for clinical practice.

To date, many reports on the prevalence of frailty in HF have been based on a unidimensional view of frailty in which only physical deficits are considered when determining frailty. An example of this view is the phenotype of frailty developed by Fried and colleagues [7]. However, the use of a measure that fails to capture all aspects of frailty (physical, psychological and social) underestimates the number of people with frailty and limits our understanding of how frailty influences cardiovascular disease and clinical outcomes [14]. Although researchers agree that frailty encompasses more than physical deficits, evaluation of psychological and social frailty is uncommon. As a result, the prevalence of psychological and social frailty in patients with HF has yet to be determined. Furthermore, although evidence supports the association between frailty and the risk of HF, to date, these associations have been based solely on physical deficits. The effects of psychological and social frailty on HF prevalence and clinical outcomes have not been established. Understanding the prevalence of different types of frailty and the effect that different types of frailty have on the risk of HF may help clinicians identify patients at risk for HF [21]. Therefore, the aims of this study were twofold. First, we aimed to investigate frailty in a sample of patients with HF from a multidimensional perspective. Second, we evaluated the association between physical, psychological and social frailty and the occurrence of HF. 

## 2. Materials and Methods

### 2.1. Study Design

The study was conducted from March 2016 to June 2019 at the Clinic of Cardiology, the Clinic of Occupational Diseases and the Clinic of Hypertension at the University Clinical Hospital in Wrocław, Poland. Patients over the age of 18 with a diagnosis of HF (all types) were recruited from these three health care facility locations (*n* = 965). Heart failure was defined according to the ESC guidelines [11]. A comparison group of patients not being treated for HF was recruited from a general medical practice at the Kosmonautów Clinic in Wrocław (*n* = 164). Informed consent was obtained from all participants. Data were collected based on interviews, observations, clinical tests and review of patients’ medical records. Interviews with HF patients were administered by a specialized nurse at the Hospital of the Wrocław Medical University, who is also a co-author of the present study. A trained family nurse collected data from participants at the general medical practice. Clinical characteristics were obtained from patients’ medical records.

### 2.2. Research Tools and Measurements

The study employed the Tilburg Frailty Indicator (TFI) to evaluate sociodemographic characteristics and frailty syndrome across physical, psychological and social domains [22]. The TFI is a 15-item self-report measure of multidimensional frailty. The overall measure of frailty ranges from 0 to 15, with higher values indicating greater frailty. Scores of 5 and higher indicate a diagnosis of frailty. Scores for subscales range from 0–8 for the physical domain, 0–4 for the psychological domain and 0–3 for the social domain. The instrument was validated in the Polish cultural setting by Uchmanowicz et al. [6]. Internal consistency reliability was demonstrated by a Cronbach’s alpha coefficient, which yielded a value of 0.74.

### 2.3. Ethical Considerations

The protocol of the study was approved by the Local Bioethics Committee at Wrocław Medical University. All patients provided their written informed consent before data collection. The investigation conformed to the principles outlined in the Declaration of Helsinki.

### 2.4. Statistical Analysis

The independent variables included sociodemographics (age, sex, education, salary and relationship status), frailty occurrence and TFI scores (physical, psychological and social subscales). For all analyses, the dependent variable was the presence of an HF diagnosis. Regression based on the probit model and chi-square statistics (χ^2^ tests) were used to examine associations between dependent and independent variables [23,24]. The marginal effects of each independent variable on HF occurrence at the mean value of the independent variable were estimated using the probit model. Next, χ^2^ tests were used to analyze HF occurrence with the TFI physical, psychological and social domains to determine if there was a significant difference between expected and observed frequencies in one or more categories within a contingency table. Robust standard errors of regression coefficients and their corresponding *p*-values were generated with each probit model. Marginal effects were also computed for the risk of HF based on the sociodemographics of interest. To avoid the effects of collinearity between the independent variables, a Belsley–Kuh–Welsch (BKW) test was performed. The probit and χ^2^ calculations were performed using the statistical package gretl (http://gretl.sourceforge.net/ accessed on 30 June 2020).

## 3. Results

The entire sample consisted of 1129 patients and healthy controls with a mean age of 69.85 (age range 18–97), and both sexes were equally represented (Table 1). Most had a high school diploma, and slightly more than half were living with a partner.

The results of the probit model examining the effects of sociodemographic variables on HF occurrence are presented in Table 2. The results showed that men were at a higher risk of having HF (15.6% increase, coefficient = 0.49, *p* < 0.001). Furthermore, we observed a 0.5% increased risk of having HF with age, indicated by a coefficient of 0.02 (*p* < 0.001). Patients in a relationship had a 11.3% lower chance of having HF (coefficient = −0.35, *p* < 0.001). A salary increase of one level indicated a 3.1% lower risk of HF (coefficient = −0.1, *p* = 0.005). For the patient subsample with frailty syndrome, sex, age and relationship status were significant. The results showed that men had a 17.5% higher risk of HF (coefficient = 0.60, *p* < 0.001). We observed a 0.4% increase in the risk of HF with age (coefficient = 0.01, *p* < 0.001). Patients in a relationship had a 9.3% lower risk of HF (coefficient = −0.31, *p* = 0.005). For the non-frail subsample, only age and salary were significant. We observed a 0.7% increase in HF with age (coefficient = 0.02, *p* = 0.004). A salary increase of one level indicated an 8.9% lower risk of HF (coefficient = −0.23, *p* = 0.002).

There was a significant association between the diagnosis of frailty syndrome and the occurrence of HF. The marginal effect indicated that frail patients had a 15.3% greater risk of having HF (coefficient = 0.39, *p* < 0.001). 

The findings from the probit models evaluating the associations between TFI domain scores and HF are presented in Table 3. For the physical domain, the marginal effect indicated a 2.9% increased risk in HF with a 1-unit increase in physical frailty based on the mean score for physical frailty (coefficient = 0.08, *p* < 0.001). The probability of HF continued to increase as physical frailty scores increased above the mean. For the psychological domain, an increase in psychological frailty of 1 unit based on the mean score for psychological frailty was associated with an increased risk of HF of 4.4% (coefficient = 0.11, *p* < 0.001). For the social domain, a 1-unit increase in social frailty based on the mean score of social frailty was associated with a 6.6% increase in the risk of HF (coefficient = 0.17, *p* < 0.001). 

Chi-square analysis revealed a significant association between physical frailty and HF diagnosis (χ^2^ = 84.49, *p* < 0.001) (see Table 4). Additional chi-square tests were performed on the dataset while restricting comparisons to adjacent scores on the physical frailty subscale (i.e., 0 vs. 1, 1 vs. 2, 2 vs. 3, etc.). Significant differences were noted when participants with a physical score of 0 were compared to those with a score of 1 (χ^2^ = 7.63, *p* < 0.01) and when those with a score of 2 were compared to those with a score of 3 (χ^2^ = 4.80, *p* < 0.05).

Chi-square analysis revealed a significant association between the psychological frailty subscale and diagnosis of HF (χ^2^ = 22.13, *p* < 0.001) (Table 5). The cross-tabulation indicated that patients with psychological frailty scores of 0 had a 49.7% chance of being diagnosed with HF. 

As with the physical frailty scores, chi-square analysis was also performed on the dataset while restricting it to differences between adjacent psychological frailty scores. There was a significant difference between participants with psychological scores of 1 and those with scores of 2 (χ^2^ = 5.36, *p* < 0.05) and between those with scores of 3 and 4 (χ^2^ = 10.88, *p* < 0.001).

Chi-square analysis revealed significant differences between the social frailty subscale and a diagnosis of HF (χ^2^ = 12.06, *p* = 0.007) (Table 6). This indicated that socially active patients (social frailty score of 0) had a 53.2% chance of being diagnosed with HF. Patients with the highest social frailty score had a 74% chance of having HF disease.

Additional chi-square tests were performed on the dataset while restricting the analysis to the differences between adjacent social frailty scores (0 vs. 1, 1 vs. 2, etc.). There was a statistical difference in HF diagnosis when those with social frailty scores of 3 were compared to those with a score of 4 (χ^2^ = 7.26, *p* = 0.007). No other comparisons demonstrated significant differences. These findings led us to conclude that the highest social frailty scores corresponded to a greater probability of developing HF.

## 4. Discussion

Our study is among the first to evaluate associations between the risk of HF and the presence of frailty from a multidimensional perspective, including physical, psychological and social domains of frailty. The findings of previous research provided support for the association between HF risk and physical frailty, and some have demonstrated that physical frailty is both a risk factor for and a consequence of CVD [15,16,17,18]. Frailty is also believed to include psychological and social components [7,14]; however, associations between the components of frailty and the risk of HF have not been evaluated previously. Reliable measures of frailty syndrome should encompass psychological and social functioning, as factors such as depression and social support have been associated with a greater risk of severe complications and poor clinical outcomes [25]. Our study builds on previous knowledge by providing evidence of an association between psychological and social frailty and the risk of HF. In fact, our study provides evidence that the risk of HF is higher given an increase in psychological or social frailty when compared to the risk of HF given an increase in physical frailty. As the complexities of frailty continue to unfold, researchers will need to continue to expand research methods to understand the mechanisms that drive the associations between frailty, HF risk and clinical outcomes.

There was a significant association between frailty and HF diagnosis in our study. Patients diagnosed with frailty were 15.3% more likely to develop HF compared to those not diagnosed with frailty. These findings are supported by previous research that only evaluated the physical components of frailty. The 11-year Health Aging and Body Composition (Health ABC) study by Khan et al. [26] found a significant association between frailty and risk of HF in a cohort of 2825 older adults. In the Health ABC study, frailty significantly predicted the incidence of HF. In addition, as many as 466 frail patients developed HF symptoms during the 11-year follow-up [26]. In addition, these researchers reported that the risk of HF incidence increased as the severity of frailty measured by the Gill index (the combined measures of walking speed and chair-stand measures) increased and that the associations between frailty and HF risk were comparable across categories of sex, age, race and clinical subgroups based on comorbid illness [26]. Similarly, a systematic review and meta-analysis by Wang et al. [27] indicated that frailty was an independent predictor of HF incidence in older patients and that frailty syndrome in this population increased the risk of mortality by 70%. Combined with our findings, the results of these studies highlight that frailty could be an independent risk factor of HF after controlling for other known risk factors. However, it should be noted that the study by Khan et al. [26] used a unidimensional measure of frailty, and the study by Wang et al. [27] included 10 studies, of which 8 used a unidimensional definition of frailty (the phenotype of frailty) [6], which limits the findings.

Our study shows that men diagnosed with frailty syndrome are more likely to develop HF than women. The results of our study are consistent with the studies published by McKechnie et al., which revealed a higher incidental HF risk among older, weak men, which persisted despite adjustment for comorbidities, known risk factors for HF and biomarkers of inflammation [28]. Age and relationship status are significant predictors of the incidence of frailty. Thinuan et al. showed that age and spouse absence were associated with a higher prevalence of prefrailty and frailty. Frailty, in turn, is associated with an increased occurrence of heart failure [29]. Salary also appears to influence the risk of heart failure. A meta-analysis by Potter et al. revealed that higher socioeconomic status (including education and income) was associated with a lower incidence of heart failure. This may be explained by the fact that patients with higher income are more likely to apply the principles of cardiovascular disease prevention and risk factor reduction [30].

Our findings demonstrating an association between frailty and the incidence of HF are supported by previous research. In the Health ABC Study, Khan and colleagues reported that a unit decrease in the HABC Battery, indicating worsening frailty, increased the risk of developing HF by 30% [26]. Kahn and colleagues further stated that excluding HF cases diagnosed in the first year of the study did not influence the risk of HF, suggesting that frailty was not merely capturing undiagnosed HF. Our findings suggested that the risk of HF due to frailty was not as great; however, different measures of frailty were used in these two studies, so this may explain the discrepancy in HF risk.

Findings from previous research support the association between frailty and mortality [4,28]. For example, Lupon et al. [4] reported that patients with chronic HF who were frail had a higher risk of mortality at one year (17% vs. 5%), higher risk of HF hospitalizations (21% vs. 13%) and lower quality of life. Similarly, Chaudhry et al. evaluated four geriatric conditions, namely, impairments in: muscle strength, gait speed, cognitive function and psychological status. Chaudhry et al. [31] showed that slow gait speed was the most powerful predictor of hospitalizations, conferring a 30% increase; weak grip strength was also predictive, conferring a 16% increase. In this longitudinal study in elderly patients with chronic HF, it was shown that frailty was an independent predictor of mortality, and the probability of survival gradually decreased as frailty increased in patients with CHF (45.5–0%) as opposed to patients without CHF (62.8% to 25.9%) [32]. Although these studies support the association between frailty and clinical outcomes, the assessments of frailty by both Lupon et al. [4] and Chaudhry et al. [31] contained few components that refer to psychological and social frailty. Future research should explore the association between psychological and social frailty and their effect on mortality.

The findings of this study have important implications for clinical practice. Clinicians should be aware that frailty is not restricted to physical limitations. Psychological and social frailty are also associated with poor self-care and higher mortality. Clinicians should routinely screen patients for multidimensional frailty using a validated measure, such as the TFI, to identify frailty at an early stage. Once identified, clinicians can address limitations in an attempt to improve self-care and clinical outcomes. Although our study indicates that frailty may be a prognostic indicator of HF, it is unclear whether or not treating frailty can reduce the risk of developing HF [17,33,34]. Although additional research is needed to explore the effects of treating frailty on HF incidence, the findings from our study support the need to evaluate frailty to ascertain the risk of future cardiovascular disease. This recommendation is in accordance with the European Society of Cardiology (ESC), the American Heart Association (AHA) and the Society for Geriatric Cardiology (SGC), which have emphasized the need to address frailty in the evaluation and treatment of patients with HF [11,35].

The present study has strengths and limitations. The study used only a single measurement for frailty assessment, which was based on the Tilburg Frailty Indicator, an extensively validated questionnaire [36]

On the other hand, we applied a multidimensional approach to frailty, which is a new approach, and evaluated frailty domains that have been previously overlooked. Frailty syndrome is still poorly understood and needs to be more clearly defined. Further research is needed to define and measure frailty syndrome adequately.

## 5. Conclusions

The findings from the present study demonstrate that frailty may be a prognostic factor of developing HF. Our study provides new evidence that psychological and social frailty are also associated with a higher risk of HF and may have a greater influence than physical frailty. Additional research is needed to compare the effects of each domain of frailty on HF and to confirm our findings.

## 6. Implication

Routine identification of frailty provides an opportunity to identify patients living with frailty, thus targeting the assessment, better coordination and planning for the delivery of interventions in patients with heart failure.

## Figures and Tables

**Table 1:** Demographics and clinic data of the sample population.

	Frailty	HF	Frailty and HF	No Frailty and No HF	Total *
Number of patients	861 (76.2%)	666 (59.0%)	562 (49.8%)	164 (15.0%)	1129
Sex	407 men (47.3%), 454 women (52.7%)	359 men (53.9%), 307 women (46.1%)	295 men (52.5%), 267 women (47.5%)	76 men (46.3%), 88 women (53.7%)	547 men (48.4%), 582 women (51.6%)
Age—mean, sdt.	71.62, 10.98	72.07, 10.97	72.88, 10.97	61.96, 11.89	69.85, 10.97
Relationship status	440 alone (51.1%), 421 in relationship (48.9%)	335 alone (50.3%), 331 in relationship (49.7%)	297 alone (52.8%), 265 in relationship (47.2%)	39 alone (23.8%), 125 in relationship (76.2%)	517 alone (45.8%), 612 in relationship (54.2%)
Education	262 basic, 106 vocational,363 high school,128 higher education	207 basic, 60 vocational,295 high school,103 higher education	190 basic, 54 vocational,242 high school,75 higher education	14 basic, 26 vocational,68 high school, 56 higher education	293 basic, 138 vocational,484 high school,212 higher education
Salary (mode)	2101 PLN or more (18.0%)	2101 PLN or more (22.2%)	2101 or more (21.0%)	2101 or more (18.3%)	2101 or more (19.0%)
Clinical characteristic	DM 316 (36.7%) HT 671 (77.9%) Both 269(31.2%)	DM 280 (42.1%) HT 513 (77.1%) Both 235 (35.3%)	DM 251 (44.7%) HT 442 (78.6%) Both 211 (37.5%)	DM 30 (18.3%) HT 141 (86.0%) Both 27 (16.4%)	DM 375 (33.2%) HT 883 (78.2%) Both 320 (28.3%)

Abbreviations: HF, heart failure; HT, arterial hypertension; DM, diabetes mellitus; PLN, polish currency (1 PLN = ~ 0.23 EUR during data collection period); * the Total column is reflective of the entire sample and is not the sum of the previous columns.

**Table 2:** Probit models for effects of sociodemographics on heart failure occurrence across the full sample and each subsample. The full sample includes participants with heart failure and those in the healthy control group. The table includes parameters, significance levels and marginal effects of the probit model built to explain effects of the five demographic variables (sex, age, education level, relationship status and salary) on heart failure occurrence.

	Sample	Coefficient	Std. Error	*z*-Score	*p*-Value	Marginal Effect
Sex	Full sample	0.49	0.10	4.89	0.000	0.1564
	Frail	0.60	0.12	5.13	0.000	0.1751
	Non-frail	0.23	0.22	1.03	0.301	0.0881
Age	Full sample	0.02	0.00	7.29	0.000	0.0049
	Frail	0.01	0.00	5.85	0.000	0.0039
	Non-frail	0.02	0.01	2.92	0.004	0.0070
Education level	Full sample	−0.04	0.05	−0.94	0.346	−0.0139
	Frail	−0.05	0.05	−1.02	0.307	−0.0157
	Non-frail	0.07	0.11	0.62	0.532	0.0261
Relationship status	Full sample	−0.35	0.10	−3.61	0.000	−0.1125
	Frail	−0.31	0.11	−2.82	0.005	−0.0937
	Non-frail	−0.40	0.23	−1.74	0.082	−0.1512
Salary	Full sample	−0.10	0.03	−2.79	0.005	−0.0309
	Frail	−0.05	0.04	−1.26	0.208	−0.0148
	Non-frail	−0.23	0.07	−3.08	0.002	−0.0890

**Table 3:** Probit models for effects of physical, psychological and social domains on heart failure occurrence. Parameters, significance levels and marginal effects of the three probit models describe the change in probability of heart failure at the mean value of the frailty subscale.

	Coefficient	Std. Error	z-Score	*p*-Value	Marginal Effect
Model with aggregate physical domain	0.08	0.01	8.88	0.000	0.0292
Model with aggregate psychological domain	0.11	0.02	5.99	0.000	0.0442
Model with aggregate social domain	0.17	0.03	6.55	0.000	0.0663

**Table 4:** Distribution of subjects by aggregate physical component index value and heart failure occurrence. Numbers in parentheses denote column-wise percentages.

		Physical Components									Sum
		0	1	2	3	4	5	6	7	8	
Heart failure	0	57 (79.2)	68 (59.6)	65 (49.6)	53 (36.6)	50 (31.6)	73 (34.3)	53 (34.6)	37 (33.0)	7 (22.6)	463 (41.0)
	1	15 (20.8)	46 (40.4)	66 (50.4)	92 (63.4)	108 (68.4)	140 (65.7)	100 (65.4)	75 (67.0)	24 (77.4)	666 (59.0)
Total		72	114	131	145	158	213	153	112	31	1129

**Table 5:** Distribution of subjects by aggregate psychological component index value and heart failure occurrence. Numbers in parentheses denote column-wise percentages.

		Psychological Components					Sum
		0	1	2	3	4	
Heart failure	0	74 (50.3)	139 (44.6)	147 (36.0)	68 (33.8)	35 (57.4)	463 (41.0)
	1	73 (49.7)	173 (55.4)	261 (64.0)	133 (66.2)	26 (42.6)	666 (59.0)
Total		147	312	408	201	61	1129

**Table 6:** Distribution of subjects by aggregate social component index value and heart failure occurrence. Numbers in parentheses denote column-wise percentages.

		Social Components				Sum
		0	1	2	3	
Heart failure	0	104 (46.8)	225 (41.0)	109 (41.6)	25 (26.0)	463 (41.0)
	1	118 (53.2)	324 (59.0)	153 (58.4)	71 (74.0)	666 (59.0)
Total		222	549	262	96	1129

## Data Availability

The data will be available by contacting the corresponding author.
